# Editorial: Advances in Microalgae Biology and Sustainable Applications

**DOI:** 10.3389/fpls.2016.01385

**Published:** 2016-09-21

**Authors:** Flavia V. Winck, Diego M. Riaño-Pachón, Telma T. Franco

**Affiliations:** ^1^Department of Biochemistry, Institute of Chemistry, University of São PauloSão Paulo, Brazil; ^2^Brazilian Bioethanol Science and Technology Laboratory, Brazilian Center for Research in Energy and MaterialsCampinas, Brazil; ^3^School of Chemical Engineering, State University of CampinasCampinas, Brazil

**Keywords:** biomass, biofuels, bioenergy, carbon dioxide, hydrogen, biotechnology, nutrients, lipids

The further development of our societies will undoubtedly face many challenges, but currently we have the capacity, and the duty, to look for environmentally sustainable solutions to these challenges. In order to do so we must dedicate efforts toward the development of processes for the sustainable production of chemicals and energy. Microalgae are receiving increasing attention as candidates to develop sustainable processes; they have been proposed as feedstock for several purposes, such as energy production, biosynthesis of nutraceuticals, animal feed and bioremediation, to mention just a few (Skjanes et al., 2013; Wijffels et al., 2013). Recent understanding of diverse aspects of microalgae biology have revealed important differences at the cellular level between them and land plants (Perez-Garcia et al., 2011; Liu and Benning, 2013). The microalgae characteristics of unicellularity and mixotrophy, are of special interest for potential industrial applications (Lowrey et al., 2016). All of the above, is positioning microalgae as a viable solution for the sustainable production of fuels and chemicals (Skjanes et al., 2013; Moody et al., 2014).

Within this Frontiers research topic, we aimed to show important advances in the understanding of microalgae biology and the opportunities to translate basic knowledge into sustainable applications.

The articles making this collection highlight how different scientific areas should come together for the successful use of microalgae biomass in sustainable applications. Here you will find articles ranging from bioprospecting regional microalgae species, through advances in microalgae molecular physiology to the development of techniques for characterization of biomass and the use of biomass into agriculture and bioenergy production.

In this special research topic, Duong et al. tapped into the biodiversity of microalgae in aquatic Australian environments, resulting in the identification of the growth and lipid content in 16 novel isolates, from five different species (Duong et al.).

The further understanding of how microalgae cells can survive on aquatic environments implies understanding the mechanisms of nutrient assimilation. This was what Sanz-Luque et al. exemplified to us in a review of nitrate assimilation and its regulation in microalgae (Sanz-Luque et al.).

Notably, the study presented in Eitzinger et al. highlighted the identification of over 40 novel proteins that make part of the eyespot, i.e., a specialized optical device, and a complex pool of methylated proteins, in *Chlamydomonas reinhardtii*, contributing to a better understanding of the primordial visual system of microalgae and its possible connection to light responses (Eitzinger et al.).

Further research revealed candidate metabolic pathways sensitive to changes on the availability of carbon dioxide, as demonstrated by Winck et al. in a genomic scale modeling approach (Winck et al.), that suggested an important role of mitochondria and enzymes of the photorespiratory pathway during cell response to high availability of carbon dioxide.

Additionally, Steinbeck et al. described a detailed study of the sustainable light-driven production of Hydrogen in the model microalgae *Chlamydomonas*. The team of researchers demonstrated the importance of double mutation of proton-gradient regulation genes for hydrogen production, with increased oxygen consumption by the mutant strains and faster achievement of anaerobiosis and activation of hydrogenases in Sulfur deprivation conditions.

Therefore, the discovery of candidate genes and biological pathways and networks can foster the bioengineering of biological routes for enhanced biosynthesis of biomass and compounds of biotechnological interest. This is shown in the article by Ouyang et al. where they performed the functional analysis of the enzyme glycerol-3-phosphate acyltransferase from the oleaginous green microalgae *Lobosphaera incisa*, revealing that alterations on the gene sequence was important for increase the enzyme activity and phospholipid content when heterologous expressed in Yeast (Ouyang et al.).

However, novel methodological approaches to facilitate the characterization of microalgae biomass have to be developed and optimized and this is what we can see in the work of Shen et al. The authors performed the global profiling of lipids in microalgae for depicting fatty acids that can be applied as characteristics ones for measurement purposes, enabling lipid assessment in several strains (Shen et al.). A detailed analysis of several processes of microalgae dewatering (which could be understood as harvesting) methods, performed by Soomro et al, discussed an important problematic in the use of microalgal biomass as feedstock; the energy and financial costs associated to the production of microalgae biomass. The authors performed a life-cycle assessment analysis and proposed a combination of selected dewatering technologies for reducing costs and increasing biomass recovery (Soomro et al.).

One example of novel applications for the sustainable use of microalgae biomass residues generated by a global future microalgae industrial application was shown by Maurya et al. The authors performed the analysis of the sustainable use of microalgae biomass residues as substitute nitrogen fertilizer on *Zea mays* (maize) cultures, showing the possibility to substitute up to 75% of the total chemical nitrogen fertilizers provided to the plants.

Well succeed applications of microalgae biomass are now on the market, such as the production of Astaxanthin by the microalgae *Haematococcus pluvialis*. Astaxanthin is a potent antioxidant molecule, and a comprehensive review of *Haematococcus pluviallis* and the many aspects of the Astaxanthin properties, production and applications was provided by Shah et al.

The production of microalgae biomass for industrial scale applications is still been developed; however, as shown by Xiao et al. large amounts of biodiesel from microalgae biomass may be produced in a controlled process.

Besides the many challenges ahead on the development of massive production of microalgae biomass it has been demonstrated that human resources are available to achieve this goal, and to improve the capacity and diversity of applications of microalgae for sustainable applications worldwide.

Scientists from different fields of research, from basic science to applied science, are indeed contributing to the understanding of microalgae diversity, their complex cellular responses and phenotypes, which will certainly contribute to generate novel solutions for the development of microalgae-based sustainable applications, which are very much needed in present times.

## Author contributions

FW draft the editorial text. FW, DR, and TF revised and approved the final version of the editorial text.

### Conflict of interest statement

The authors declare that the research was conducted in the absence of any commercial or financial relationships that could be construed as a potential conflict of interest.
